# Pain

**Published:** 1903-08

**Authors:** Charles Gilbert Chaddock

**Affiliations:** St. Louis


### PAIN.[1]

BY CHARLES GILBERT CHADDOCK, M.D., ST. LOUIS.[2]

GENTLEMEN,—Allow me first to express a deep appreciation of the honor accorded me in the request to lecture to you.

Since my daily work is mainly in the domain of the nervous system, you will expect that anything I have to present will be decidedly colored by my usual manner of thought, and you will be in no way surprised that I have chosen *pain* as a very absorbing, if not interesting, matter for discussion.

---

[1] A lecture delivered to the students of the Marion-Sims Dental College, March 13, 1903.

[2] Professor of Diseases of the Nervous System in the Marion-Sims-Beaumont College of Medicine, Medical Department of St. Louis University.

People come to you most frequently because they have pain, and very often they must endure more at your hands before they feel less; they are not all like those lucky (or shall I say unlucky) ones that are cured of the toothache an instant before they push the button of the dentist's door-bell. Pain is no joke, no matter how ludicrous some of its vagaries may be, when such vagaries have some one else for a plaything; here I propose to consider it as a serious thing, no matter how much you may be inclined to ignore it; for if the consensus of opinion is unanimous on any one point it is on the fact that dentists are the most excruciatingly painful operators that the science of medicine has evolved.

Pain must be considered, if you would understand it, from a physical and a mental stand-point. We speak of physical pain and of mental pain. Physical pain is that dependent upon some tangible physical cause; mental pain is the pain attending a state of mind (an emotional condition). But there is another kind of mental pain, that presents all the characteristics of a true physical pain, for which no physical cause can be found. The painful emotional state known as psychalgia I shall not consider here, for it belongs to the symptoms of mental alienation (insanity). The true physical pain and the mental pain resembling it are the two phenomena to which this brief discussion must be limited.

It is impossible to consider pain from merely a physical view-point; for pain is a quality of general sensation, and sensation is one of the attributes of the mind which cannot exist save as a part of mind.

In parenthesis, I would reply to any objections to this statement by saying that reflex activity excited by irritation of a sensory nerve,—as the movement of a decapitated frog's leg to brush from the flank a particle of paper moistened in acid,—though a manifestation of mind in the broadest sense, cannot be proved to be a conscious act, and therefore cannot be considered as necessarily attended by sensation.

Sensation is a subjective phenomenon,—*i.e.*, we can know nothing of what another feels except indirectly. A person tells you he is experiencing pain, and acts in a way to convince you that it is a fact of his inner subjective experience; you may be convinced by his acts alone that he is feeling pain, for your own acts under like circumstances of subjective experience have been similar. Thus, you will understand how we can know nothing of what another feels except through indirect testimony. For the practical purpose of this lecture, I would limit mind to its manifestations in human beings,—manifestations which we are able to study through our own parallel and similar subjective experiences,—and thus you will accept, in this limited sense, the statement that sensation is an attribute of mind depending on consciousness for its existence. Then the real seat of pain is invariably the cerebral cortex, upon the existence of which consciousness depends—where perceptions arise.

We say, without exception, that we have a pain here or there in the body, and this is theoretically correct, and practically important, but actually we have pain only in consciousness which is referred to one point or another either for physical or mental reasons. You will understand this better by a concrete illustration: A patient has an excruciating trigeminal neuralgia; if the surgeon sever the sensory root-fibres between the Gasserian ganglion and the pons varolii, the pain ceases if the cause of it lay in the ganglion or any of its three branches; but the ganglion and its branches remain and present the same physical condition as before, and therefore the seat of the pain was not there, but higher in the central nervous system (cortex). A man has lost a limb by amputation, but continues to experience pain and other sensations in the lost part,—another conclusive demonstration that the seat of sensation is not in the limb, but in the central nervous system. These two illustrations serve to emphasize the practical difference between pain caused by an external condition (physical pain) and pain due to independent cortical activity (psychic) and *referred* to some peripheral part of the body.

It will be well to remind you by other familiar facts of the predominating *rôle* of consciousness in the aspect of sensation known as pain. You will recall that the general anæsthetics, chloroform, ether, etc., exercise their beneficent effect through their power to produce unconsciousness of a degree so profound that external stimuli fail to arouse the functions of the cerebral cortex. Other material agents have a similar influence; morphia, for example, has a certain selective effect, whereby it overcomes pain through its double influence on the central and peripheral nervous systems. And cocaine and allied drugs act similarly both locally and through the circulation.

The most powerful means of subduing pain is by reducing the impressionability of the cortex of the brain, and it is to this aspect of the subject that I shall give most attention, for it is the side most frequently forgotten in our every-day dealings with pain. Before alluding to means of affecting the state of consciousness as related to pain, allow me to call your attention briefly to those portions of the nervous system that are known to be concerned in the origin of general sensibility in its various forms.

General sensibility presents several qualities, among which touch, pain, and the sense of temperature are the most important. It is highly probable that there are distinct peripheral organs, anatomically differentiated, for these three forms of general sensibility. There are organs called tactile corpuscles, and their functional relation to the sense of touch is proved; however, they may also be simultaneously concerned in the conduction of stimuli that give rise to perceptions of pain and changes of temperature. Certain it is that the sense of touch does not depend on the tactile corpuscles alone. I have never heard of such organs in the teeth; but the teeth have a very delicate impressionability to touch, and that independently of any mechanical stimulus arising from movement of the teeth in the tissues in which they lie partially embedded, as you may prove yourselves by experimentation with a feather. You all know, too, that the teeth enable us to appreciate very delicately changes of temperature; they are also quite well known as performing a part in the generation of pain. From this we may conclude that if the terminal organs of sensory nerves serve special functions within the domain of general sensibility, they do not necessarily lose any of their fundamental relations to the three qualities of general sensibility.

Though the nerve-endings may be differentiated for these three kinds of sensation, we do not find differences in the peripheral nerves to serve for their conduction. In a mixed nerve we cannot even distinguish anatomically between the motor and sensory fibres. In the spinal cord the state of our knowledge is much the same, though generally accepted views here distinguish certain distinct pathways for the conduction of certain forms of sensory impressions. It is practically certain that the distinction between sensory and motor nerves, so absolute in the composition of the anterior and posterior nerve-roots of the spinal cord, is maintained in the internal arrangement of the conducting paths in the cord. The motor elements of the spinal cord make up but a comparatively small mass of its aggregate of matter, and they all lie in the lateral and anterior portions of the cord; the sensory or in-conducting elements of the cord are made up of the posterior columns, the greater portion of the gray matter, and a large portion of the lateral columns of the spinal cord. Theoretically we can comprehend this preponderance of nervous matter devoted to sensory functions when we consider for a moment the relative needs of these two aspects of mind. Complicated as our movements are, they are much less complex considered with respect to their motor aspect than with respect to their sensory side; for all possible movements are the results of the activity of a limited number of muscles, while the innumerable combinations and adjustments of muscular actions depend upon a corresponding development of the elements that serve sensation. Muscular co-ordination, equilibrium, the sense of position, etc., depend on an infinite variety of sensations which must have numerous avenues and centres interrelated and associated that subserve them. A glance at the functional areas of the cerebral cortex shows you that only a comparatively small area is concerned directly in motion, while the sensory areas comprise the motor and many other regions of the cortex and brain (temporal, parietal, occipital lobes, cerebellum, basal ganglia), an additional demonstration of the predominance in mass of the sensory elements over the motor elements of the nervous system.

The sensory paths in the spinal cord we need not consider here in much detail. Those for the sense of touch are said to pass upward in the posterior columns to the medulla, where they cross the median line and, continuing their course farther, reach the optic thalami, whence they proceed farther through the internal capsule, intermingled with down-conducting motor fibres, to end in the motor areas of the cortex. Whether the sense of touch depends on impressions travelling exclusively by the route just traced is still an open question; however, it seems to be quite conclusively established that the principal upward pathway for general sensibility is the *central gray matter* of the cord, with the connected great ganglia of the base (optic thalami), and the fibres that associate these with all parts of the cerebral cortex. Destruction of the central gray matter of the cord, especially the posterior portion of it, causes loss of capacity to perceive painful and heat impressions in corresponding areas of the surface of the body, while in such cases the sense of touch may remain quite unaltered. Thus there seems to be in the cord a differentiation of sensory paths that we can find no trace of in the peripheral nerves;[1] but here this apparent differentiation is not conclusively demonstrated, and all we can safely say is that the main pathway of general sensory impressions in the central nervous system is the spinal gray with its associated basal ganglia and their connections with the cortex, mainly the central convolutions.

I have thus traced for you the route by which an impression travels to the organ of consciousness, where, if it possess a certain quality, it is recognized as painful.

It is a law applicable to peripheral sensory nerves at least, that irritation of its trunk causes sensations that are referred to the seat of the irritation and to the area of peripheral distribution of the sensory filaments of the nerve as well: when you strike your funny-bone you hurt your elbow and have a painful sensation in the little and ring-finger. This reference of sensation to the distal periphery from a proximal physical cause also happens in irritation of the sensory nerve-roots of the cord, as is seen in lesions of these roots in locomotor ataxia and meningitic processes that implicate them locally, where the distribution of pain corresponds with the areas supplied by the nerves that are gathered together to form the roots affected. In the early stages of transverse lesions of the cord we observe often enough the same phenomenon in the "pins and needles" sensation that is felt in areas below the level of the lesion. In cases of cerebral disease implicating the internal capsule or the cerebral cortex of the motor areas, often the precursory symptoms are abnormal sensations referred to the opposite side of the body,—not usually pains, but still unpleasant sensations. (Why pain is not felt under such circumstances I shall discuss presently.) We have thus every reason to believe that this law of reference of pain or sensation to the periphery is applicable to the whole length of the sensory pathway.

Irritation of a peripheral nerve or of its ganglion gives rise to pain referred to the corresponding peripheral area, and irritation of the sensory pathway in the central nervous system in the cord or at its termination in the motor area of the cortex causes

---

[1] The rare dissociation of sensibility in cases of peripheral neuritis does not demonstrate a differentiation of sensory nerves for the exclusive conduction of the various modes of sensibility.

sensations likewise referred to the corresponding peripheral areas, but these sensations have not the characteristic quality of pain. Therefore it would seem that the peripheral sensory cell of the ganglion of the posterior spinal root and its peripheral prolongation (the sensory nerve) have some specific form of reaction to external stimuli which, conducted centrally, is felt as pain, while the central pathway reacts to local irritation in another way felt as alterations of normal sensations,—not pain, but referred, nevertheless, to the corresponding peripheral areas. It would seem that the sensory nerve-cells of the peripheral ganglia (ganglia of the posterior roots and their morphologic equivalents of the cranial nerves) have the power to intensify (multiply) peripheral irritation and thus send the impression inward to the perceptive centres, while irritation of the same kind affecting the central elements of the sensory pathways has not this power, but gives rise to disturbances (mainly reduction) of sensibility that are rarely if ever felt as pain.

Thus the peripheral nerve and its cell in the ganglion of the posterior root and the receptive (perceptive) centre of the cortex of the brain are the two elements of the sensory nervous mechanism essential for the development of perception of a painful sensation; the intermediate links between them are essential as conductors, but not as generators, of painful impressions. Allow me to illustrate my meaning by reference to a mechanico-electrical contrivance: A telephone is made up of two instruments and a connecting circuit of wire; the two instruments are absolutely the determining elements of the nature of the sounds transmitted; an electrical current originated in the connecting circuit is transmitted, to be sure, but has no specific effect on the receiving instrument. Remember that through a telephone you have no difficulty in recognizing a well-known voice by certain qualities. You cannot conceive that such distinguishing qualities of the voice are separately transmitted over a wire; you know, rather, that the interruptions produced by a voice in the transmitter are reproduced, through the medium of the connecting circuit, by an instrument exactly like the transmitter,—the receiver,—so that the sounds that affect one instrument are not transmitted, but actually recreated in the other.

From this it must follow that there is a possible creation in the central organ of perception of that which corresponds with an external or peripheral event; and it follows, from the law of reference to the periphery (the external or objective world) of all that takes place in the internal sphere, that such creations are, if they possess sufficient intensity, always interpreted as of external origin.

A peripheral sensory nerve may conduct impressions indifferently in either direction, as is shown by the well-known experiment of engrafting the tip of a rat's tail in the skin of the back and severing it at its root, after which sensory impressions are conducted from the root of the tail towards its tip, which has developed new nervous connections at the point where it has been engrafted. Thus we may consider it probable that a reproduced sensory impression originating in a cortical centre may travel outward over its corresponding sensory pathway, and so affect the peripheral organ—ganglion and peripheral nerve—as to excite there a specific reaction like that excited by actual mechanical stimulation, and which, reconducted to the cortical centre creates the nervous condition that normally arises from actual mechanical irritation of the peripheral organ (ganglion and nerve).

You may consider this physiologico-psychologic discussion to be extremely wide of the mark, but you will grant me the privilege of presenting a theory before exposing facts that would be inexplicable without this introduction. I mean to say that the preceding considerations, though they may not offer a demonstrably true explanation of some facts of nervous physiology, still give us a working basis for a more or less satisfactory understanding of certain nervous phenomena that we meet every day.

Please allow me to proceed a step farther in psychology before presenting some practical examples of the importance of comprehending the physical and psychic aspects of pain. Let us consider consciousness and what is known as attention. Consciousness is a term used to designate our recognition of sensations, perceptions, feelings, volitions, and the higher intellectual operations that take place in us—in our minds. We speak of the field of consciousness, just as we do of the field of vision, for the number and variety of sense and mental impressions of which we are aware at any one moment of time vary extremely. Thus the mind may be exclusively occupied with a very few things, even a single thing, or alive to a great number of things. The field of consciousness is enlarged or contracted in accordance with what we call attention. Attention, as we commonly conceive it, is the voluntary limitation of our field of consciousness to any given number of sense impressions or ideas. It is a fact of every-day experience that we have this voluntary power, within certain varying limits, to increase our consciousness of some and decrease our consciousness of other mental activities; but we have all also learned that there are certain forms of sense impressions that, in spite of all voluntary effort, rush into consciousness and insistently and against our wills absorb attention to the exclusion of that of which we may wish to think. Thus there are two aspects of attention as a quality of consciousness: it may be the immediate result of volition (our ordinary manner of regarding it), or it may be our attention is forced to concern itself with certain mental phenomena. This distinction of voluntary and forced attention is of capital importance as a basis for explanation of the vagaries of pain, and requires some further consideration. If we abandon ourselves to a spontaneous state of consciousness, it will be noted that attention wanders from one sensory domain to another, or from one train of thought to another, always guided by or absorbed by the most intense or insistent impression or idea; then, if at any moment some idea or impression excite a pleasurable painful feeling, we may experience a spontaneous awakening of voluntary attention and be induced to pursue it indefinitely to the exclusion of all other mental impressions that a moment before seemed to have an equal chance of winning attention. Thus within certain limits the play of involuntary attention is determined by the intensity of sense-impressions, passing from the less to the more intense. With a normal and moderate intensity of sense-impressions the will may always determine those that shall enter consciousness, and what ideas and trains of thought shall occupy consciousness. As generally educated and trained we have, however, a very limited control of intense sense-impressions, and are decidedly their slaves when they have for any reason become unusually intense. For example, if one be intensely absorbed in study, all ordinary and moderate sensory impressions pass unperceived: one suddenly awakes to find that he has become thoroughly chilled in a cold room, but the chilling process took place so gradually that its intense degree was necessary before it could change the direction of attention dictated by the will. Under like circumstances a loud cry of fire would immediately change the direction of attention and banish the whole voluntarily awakened train of thought. Perhaps the most common and effectual disturber of voluntary attention is pain. Some persons can go about their daily occupations when in pain, but we can conceive a pain so intense that attention can be given to nothing else. Ability to employ the mind at will while in pain is determined by two factors: sensibility to pain varies with the individual, and the voluntary power to render one's self more or less oblivious to pain varies with education and training. Obliviousness to pain, or to impressions that would usually cause pain, may be due to other involuntary mental states, especially violent emotions, which in themselves are most powerful in limiting or narrowing the field of consciousness to perceptions and ideas in harmony with them. In the indescribable excitement of battle wounds that would be painful go unnoticed until the excitement has subsided, when they claim the undivided attention of the sufferer, and even artificial means in such cases may prove ineffectual in overcoming it, when before a simple state of mind was sufficient to prevent perception of pain.

You will all recall the experiment of boyhood, when you were told that a pin could be plunged in your calf without causing a painful sensation, and the experimenter hit you a sound rap with a lath and was successful in rendering you oblivious to the prick while you had not yet had time to recover from the sting of the blow. There could be no more simple demonstration of the power of one intense impression to obliterate or prevent the occurrence of another of less intensity. In its essence the principle here involved of the prevention of a pain by a pain more intense, is the same as that which is operative for any intense state of consciousness as opposed to one of less intensity. For the same reason concentration of thought (imagination) from emotional causes may be so intense as to preclude the entrance into consciousness of anything not in harmony with the dominant emotional state and accompanying ideas. You have but to recall the celebrated examples of religious ascetics, who in fasting, prayer, and immobility on the tops of columns remained indefinitely oblivious to the pangs of the flesh; of others who maintained a given posture of supplication or prayer until so fixed by tissue changes that movement became next to impossible; of religious martyrs who found joy in what would be pain to another. Such examples of voluntary or involuntary experience of pleasure in that which could only cause pain in ordinary persons are simply examples of emotional limitation of the field of consciousness to pleasant sensations to the exclusion of pain. Such a state of consciousness may even go so far as to cause impressions normally painful to become pleasant. In some parts of Russia the knout is one of the essential instruments of the household,—even more necessary than kitchen utensils; for the wife finds joy in the lash administered by the husband as a proof of his love. If these Russian women complain of marital neglect, it is because their husbands have not shown sufficient interest to give them a few telling stripes with the whip.

Pain, then, may be influenced by the intellect, the emotions, or the will; it may be eliminated from consciousness when there is an ordinarily efficient cause for it, or it may be created when there is entire absence of any physical cause for it. Allow me to cite a few examples from literature and my own experience which the previous discussion I hope will serve to explain.

"During the year 1862 I was called upon to give chloroform to a very nervous and highly hysterical girl, who was about to have two sebaceous tumors of the scalp removed. It was found that there was no chloroform at hand. I took the inhaler, which had no odor of chloroform. Having sent for the chloroform, I placed the inhaler over her face to accustom her to it. She at once began to breathe rapidly through it. In half a minute she said, 'Oh, I feel it; I feel I am going off;' and, as the chloroform had not yet arrived, she was told to go on breathing quietly. At this time her hand slipped down by her side and I pinched it, and again harder and as hard as I could, and to my surprise she did not seem to feel at all. Finding that this was the case, I asked the operator to begin, and he incised one of the tumors and pulled out the cyst. At this time I had removed the inhaler, and, wishing to see the effect of her imagination, I said to the operator, who was about to remove the second tumor, 'Wait a minute, she seems to be coming round.' Instantly her respiration, which had been quiet, altered in character, becoming rapid, as when I first applied the inhaler, and she commenced to move her arms about. I then reapplied the face-piece and the second tumor was removed. The patient felt no pain and was unconscious of all that was done."

"Exactly ten years later, that is, in 1872, I met with a similar case. On June 16, 1872, Kate Levy, aged twenty, came to the Dental Hospital of London to have some carious teeth extracted, but, as more than one sitting was deemed necessary, it was proposed to remove the two most painful and difficult teeth, and then order her to come again. This patient, like the last, was of a very hysterical temperament, but had nitrous oxide administered without any difficulty, and the extractions were performed; when she came to herself she refused to sit up in the chair or to push the piece of wood from between her teeth (where it had been placed for the purpose of keeping her mouth open), that is, she remained perfectly motionless, not taking any notice of surrounding objects, and not doing anything she was told to do. That she was conscious I knew by the expression of her face, by the quivering of both eyelids, and by the reflex action which immediately ensued on touching the conjunctiva. I therefore said to the operator, 'Well, if she is still unconscious there will be time to remove another tooth,' for eleven teeth and roots had to be extracted, and, to my surprise, this was done without any apparent suffering. I then remembered the previous case, and said to the operator, 'Now she is coming round.' She thereupon opened her eyes, sat up, and recovered in the usual way that patients do. She came again on June 21, and, wishing to try the influence her imagination had on her sensibility, I determined to administer air only. She breathed this for a few seconds, and on my calling the students' attention to her mode of quiet breathing, she began to inhale the air more deeply, and then, on my giving her the cue by saying to the students present, 'Now, you see, when I lift up her hand, and then let it go, it will fall heavily,' it turned out as was predicted; then saying, 'Now, you will find she will breathe rapidly and then cease to feel pain, although she will know something is being done,' I removed the face-piece. The operator then commenced his extractions, removed four teeth, and in the hospital note-book it is written: 'The patient breathed air only through the inhaler; one firm tooth, two firm stumps, and a temporary tooth extracted; said she felt no pain, but felt the teeth coming out.' "[1]

These examples are clear demonstrations of the effect of idea to prevent pain.

Quite as interesting from a practical point of view is the influence of idea to cause pain. I believe any of you can experiment on himself in such a way as to satisfactorily demonstrate this possi-

---

[1] Illustrations of the Influence of the Mind upon the Body, D. H. Tuke. H. C. Lea's Son & Co., Philadelphia, 1884.

bility. If one concentrates his attention on any given part of the body, in a short time he will begin to perceive sensations in that part which previously did not exist, and they will be found to come and go with the direction of attention to or from the part, and a pain may be thus created. Dr. Tuke[1] cites a case related as occurring in the year 1607, of a parson's wife in England who consulted her physician, and was told that she probably had sciatica. Thanks to the idea implanted in her mind, she developed sciatic pain the same night, though before seeing her physician she had had no symptom of sciatica. This is an early example of unintentional creation of a symptom by suggestion, a proceeding that has been very cunningly utilized by a certain class of modern charlatans who claim to possess special powers of divining the nature and symptoms of disease in a patient without any description of symptoms by the patient. I need but sketch the manner of procedure: the operator, if she—for many such are women—by some evident sign is unable to guess the part in which the patient suffers, proceeds cautiously to palpate and adroitly learn from the patient the seat of trouble; but failing in this, she boldly asserts that the patient is subject to this or that symptom, and discourses learnedly about causes and means of cure. If the patient be a matter-of-fact person, the trick fails; matter-of-fact persons, however, rarely consult such charlatans; their clientele is composed of impressionable nervous persons, who come believing and with an all-embracing faith, and leave with the conviction that they have the aches and pains that have been suggested to them, provided they have not inadvertently and unconsciously revealed any malady with which they may be suffering. Such miraculous powers of divination engender a similar faith in curative power, and in the great majority of cases they are benefited, and often cured if the symptoms happen to be of a functional (mental) kind.

I recall a remarkable instance of ideational pain that came under my observation in a certain nervous clinic in Paris. Two girls in their twenties came for the same trouble, presenting identical symptoms, both complaining of excruciating pains in the lower limbs, of girdle pains around the trunk, and both walked with an ataxic gait. These patients occupied the same room; one had developed these symptoms, and a short time later they appeared

---

[1] Op. cit.

in the other. The patient that first developed them was found to present all the objective signs of locomotor ataxia; her companion had developed all the subjective symptoms of the disease and had imitated the ataxia, but presented none of its objective symptoms; she had developed them by imitation (idea). One had an organic disease of the cord; the other was immediately cured of all her symptoms by the assurance that nothing whatever was the matter with her beyond a too lively imagination.

Another very frequent cause of psychic pain lies in experience of physical pain. Many impressionable persons, as a result of trauma or actual disease processes, have pain in some part of the body. Sooner or later the physical cause of this pain is removed or disappears; the pain, however, continues indefinitely after the removal of its original cause, and is thus purely an ideational pain, the reason for the continuance of which lies solely in the conviction that the original physical cause is still acting. I might cite many examples of this kind from personal experience. I feel sure that dentists often meet cases in which the teeth remain sensitive and painful after all physical cause for such a condition has been entirely removed. As an example of this condition, I recall a man in his fifties, a laborer, who injured his right arm while using a shovel, and was obliged to abandon work for some time. When he tried to resume work he was unable to do so because of pain in his arm. This incapacity had continued for a year, when he came to the clinic for advice. All his symptoms were subjective, and he was told that a few applications of electricity would cure him. In two weeks he was working as before.

The emotion of fear coupled with idea is a most potent cause of purely psychic pains. You are all, doubtless, familiar with the fact that many students of medicine imagine that they discover the symptoms in themselves of the diseases they are studying. I recall the case of a young doctor who was quite convinced that he was developing locomotor ataxia because he was brought into contact with many patients suffering with that malady. He had pains and disturbances of sensibility in the legs and the ulnar borders of the arms and hands, a girdle sensation, some disturbances of the functions of the bladder, and even thought he detected some ataxia. The interesting feature of this case was that though rationally convinced that all these symptoms were imaginary, they continued to trouble him for a year or more after, the abnormal sensations obtruding themselves in consciousness suddenly when he was occupied intensely with other matters. The psychology of this case seemed to be that fear of a possibility had engendered a habit of attention to bodily sensations which were at first interpreted as proof of the oncoming of ataxia, and that this habit continued after the intellectual conviction of its imaginary nature had been established, so that for a long time any slight sudden pain had power to evoke the old fear and consequent attention to sensations having no significance whatever, and which only a strong effort of the will could banish.

A more direct example of pain due to fear is that of a butcher who in the effort to hook up a piece of meat slipped and became suspended by the arm on an adjoining hook. When taken down he was suffering agony, but examination showed his arm to be uninjured; he had been suspended by his coat-sleeve. There is a remarkable case related of a distinguished physician who was able at will to produce a more or less severe pain in almost any part of the body.[1]

Such examples might be indefinitely multiplied, but I think I have cited enough to make it clear to you that the mind exercises a most profound influence on bodily sensations.

Expectation of a sensation greatly enhances its intensity, and for this reason we feel pain more acutely if we expect it. Doubtless many of your patients thus suffer more at your hands than they would were they not dreading the ordeal of your ministrations.

Before concluding this lecture with some practical hints with reference to your attitude towards patients and to the distinctions between psychic and physical pain for purposes of diagnosis, I wish to add a few more striking examples of the effect of the mind to cause or remove abnormal symptoms. Dr. John Brown, of Edinburgh, wrote a prescription for a laboring man, telling him to take that and he would come back in a fortnight well. He returned at the appointed time well and hearty. Struck by the success of the medicine prescribed the doctor looked for a record of what he gave him; but the man said, "Oh, I took it, I swallowed it,"—the paper.[2]

The following case is an illustration worthy of comment from several points of view: "An event in the life of Andrew Crosse,[3]

---

[1] Tuke, op. cit. [2] Ibid. [3] Ibid.

the electrician, illustrates in a striking manner the power of the will over threatening disease. In this case the symptoms were those of hydrophobia. Mr. Crosse was severely bitten by a cat that died the same day of hydrophobia. He appears to have thought little of the circumstance, and was certainly not nervous or imaginative in regard to it. Three months, however, after he had received the wound, he felt one morning great pain in his arm, accompanied by extreme thirst. He called for a glass of water. He describes his experience in his own words: 'At the instant that I was about to raise the tumbler to my lips, a strong spasm shot across my throat; immediately the terrible conviction came to my mind that I was about to fall a victim to hydrophobia, the consequence of the bite that I had received from the cat. The agony of mind I endured for an hour is indescribable; the contemplation of such a death—death from hydrophobia—was almost insupportable; the torments of hell itself could not have surpassed what I suffered. The pain, which had first commenced in my hand, passed up to the elbow and from there to the shoulder, threatening to extend. I felt all human aid was useless, and I believed that I must die. At length I began to reflect upon my condition. I said to myself, either I shall die or I shall not die; if I do it will only be a fate similar to that others have suffered, and many more must suffer, and I must bear it like a man; if, on the contrary, there is any hope of my life, my only chance is in summoning my utmost resolution, defying the attack, and exerting every effort of my mind. Accordingly, feeling that physical as well as mental exertion was necessary, I took my gun, shouldered it, and went out for the purpose of shooting, my arm aching the while intolerably. I met with no sport, but I walked the whole afternoon, exerting at every step I took a strong mental effort against the disease. When I returned to the house I was decidedly better; I was able to eat some dinner, and drank water as usual. The next morning the aching pain had gone down to my elbow, the following it went down to my wrist, and the third day left me altogether.[1] I mentioned the circumstance to Dr. Kinglake, and he said he certainly considered that I had an attack of hydrophobia, which would possibly have proved fatal had I not struggled against it by a strong effort of mind.'" ("Memoirs of Andrew Crosse," p. 125.)

---

[1] This is distinctly descriptive of hysteria.

I must humbly differ with the opinion of Dr. Kinglake concerning the nature of the case. This, to my mind, is a case exactly like that of the young physician who feared ataxia. The gentleman probably had a transient attack of gout, which he walked off. By chance he felt a pain in the hand that had been bitten, and this awakened all the other supposed symptoms of hydrophobia. However, if Mr. Crosse had not exerted his will, as Dr. Kinglake said, he might have died of the disease he imagined he had. I have no doubt that cases of mental hydrophobia occur and end fatally; and in epidemics of a terrible kind, like cholera, doubtless the imagination, excited through terror, claims many victims, and that independently of infection. In support of this allow me to quote a case of Dr. Lisle:[1] "This physician recognized the utility of the imagination in the treatment of disease, and was in the habit of prescribing two kinds of silver-coated bread-pills; one kind was invested with violent purgative qualities by its name only. He had under his care a man who believed himself to be afflicted with a most obstinate form of constipation, and one day he told this patient that he would give him a most violent purgative, which would certainly render him very ill. The patient was ordered to take five of these silver-coated bread pills, an interval of a quarter of an hour between each. After the third pill the patient was well purged, and in seven hours the bowels were acted upon more than twenty times. He was jubilant at the successful operation of this new purgative, but was almost in a state of collapse."

There could be no more striking example of the effect of the imagination upon the organic functions of the body, and I believe it fully supports my contention of the gravity of fear as inducing even fatal disease.

You will note that in this discussion I have made no reference to hypnotism as a means of influencing pain. I have purposely avoided it, because the time at my command will not permit more than allusion to it. Hypnotism, in fact, is nothing more than an intensification of the suggestive ideational influences that we employ daily in our treatment of patients,—to create mental states, or to modify those that may be present.

I wish to conclude this lecture by some practical deductions that are the logical sequence of what I have attempted to present.

---

[1] Tuke, op. cit.

Since your calling is one that almost necessarily causes pain, seek to understand the temperament of your patients and try by adroit influence to lessen their susceptibility to pain, both by diverting their attention and by resort to innocent methods of deception. Your own observation will teach you to know that you must approach and treat patients individually; you will learn that the frank and positive assurance of painlessness to follow some futile application is often productive of satisfaction to the patient and renown to the dentist. Timidity or lack of assurance in your attitude is almost sure to cause an antagonistic and unprofitable state of mind in your patients. I think you should never rely on the known anæsthetic local effect alone of certain drugs, but always seek to increase it by assurances of their efficacy. Of course, there are persons little open to suggestive influence, but you will soon learn to distinguish them from the great multitude of those open to suggestion. You will also learn that the suggestion in itself is often of little value; the operator—his personality and manner of approaching a timid and nervous patient—does much to render his "suggestion" effectual.

Another practical point that demands consideration is the importance for the welfare of the patient of distinguishing psychic pain from pain having an adequate physical cause. You have already seen that suggestion (idea) is effectual in both kinds of suffering, and that simply because by ideational influence we are able to abolish pain, we are in no wise justified in assuming that there was no physical cause for pain. Your office is to treat and remedy physical conditions often causing pain, and, however expedient any means of abolishing pain may be, your ultimate purpose is to remove any physical cause there may be for pain; and it is your most important duty to determine whether a given pain seemingly arising from the teeth is actually caused physically or is merely psychic. Are there means of deciding this question? I think there are in the vast majority of cases. Of course, when you find evidence in the condition of the teeth for toothache or facial neuralgia, your course is plainly indicated; it is in cases where there is a doubt that care must be used before resorting to operative interference. There can be no question that pathologic conditions of the teeth are very frequently the cause of facial neuralgias the seat of which is not obviously in the teeth, and in such cases the most care is necessary. The temptation to resort to extraction of teeth when no other cause for facial pain can be readily discovered is very great, and I would caution you against yielding to it. If you as dentists can find no cause for pain in the teeth or jaws, it is your duty to shift all responsibility for extraction of the teeth upon some one else,—the patient's medical adviser or the patient himself; and if you are convinced that the teeth are not at fault, you should even refuse to be a party to an operation for which you can find no reason. You will remember that pain about the head, face, and teeth may be due to many causes. Take, for example, trifacial neuralgia; it may be due to disease of the nose, to disease of the antrum, to disease of the ear, to some infection like malaria, to disease of the Gasserian ganglion, to a psychic condition. There is not time to discuss the differential diagnosis of all these possible causes, but I would say that you can easily and practically define your own position and responsibilities in a given case by limiting your diagnosis to the conditions found in the teeth and jaws. Since, however, I have emphasized the psychic aspect of pain, you will permit me, in conclusion, to enlarge upon its differential diagnosis from pain due to physical causes.

Physical pain obeys certain laws in its manifestations that aid us in deciding the doubtful case. I cannot go into them all, for I have already taxed your attention and patience unduly; but you will bear with me a few moments longer. Mechanical irritation of a nerve-territory that is already the seat of physical pain is sure to increase the pain arising in it, but when pain in a given area is psychic, mechanical irritation in it does not produce identical effects. With actual physical pain, the increase of pain is in direct proportion to the degree of direct physical irritation; with psychic pain the increase of pain from direct physical irritation of the area involved is not proportionate to the degree of irritation,—that is, slight physical irritation produces a painful effect equal to that which follows severe irritation. In actual organic disease of the nervous system there are found some exceptions to this, as in the hyperæsthesia sometimes observed in locomotor ataxia; but in such conditions the areas implicated enable us to refer it to an anatomical cause—to nerve-roots or nerves that control definite areas. Thus a pain limited to a certain known distribution of a sensory nerve or nerves is almost certainly due to a physical cause; for psychic pain can be limited to the distribution of a nerve only when the individual affected is acquainted with the distribution of nerves. Another point: where small areas are concerned, the limitation of the area is clearly marked in case the pain be due to an actual physical cause; whereas, a psychic pain is more apt to be indefinitely limited in relation to the area from which it may be excited or increased in intensity by mechanical irritation; that is, the superficial area to which a psychic pain is referred varies extremely during the same examination and on repeated examinations.

Again, to refer to the teeth directly: you have all, perhaps, found some difficulty in locating an abscess at the root of a tooth because more than one tooth was painful to irritation, but you have usually been able to locate the trouble by finding the most painful spot on the gums or on the jaw. Psychic pain about the jaws would lead you astray because of the absence of definite localization.

I would modestly suggest, as one only speaking from a very general point of view, that you proceed with caution in your operative interference when you have not definite facts upon which to base your action, remembering always that the teeth are very frequently not the cause of pain that is referred to them.

In your observation of patients of all classes and of all conditions of life you will notice certain peculiarities that will strike you as remarkable. In the long run, if I may venture to foretell one thing, you will be surprised by a difference between your country and city patients: country patients, in a sense, will endure pain better than city patients; I say, in a sense, for the reason that the man who lives close to nature and is in a robust physical condition from a closer relation to the elements of nature is less sensitive to pain than his more highly and artificially organized brother that has been reared and lives in the city. But if the country patient is less sensitive to pain he is at the same time less able to endure it than his city cousin. It is well known that city-bred men make better soldiers at first than country-bred youth; the former have more endurance and bear hardships better. Thus you will observe among country-bred patients that they will readily endure an extraction before which a city patient would quail; a city patient, to avoid an extraction, would endure a thousand times more pain in treatment or arrangement of a tooth, while the country patient would say, pull it out and let's have an end of it. In the one case fear of pain cultivated by reading and example renders more sensitive, while endurance of it is strengthened by the will to have a cosmetic effect for which the other does not care.

Pain is not an unmixed evil: it has been proved that the brutality of the cruel criminal is in direct proportion to his lack of sensitiveness to pain. Our sympathy with the distress of others is in direct proportion to our sensibility to pain.

If it be necessary for me to urge you to save teeth, I need only remind you, from a selfish stand-point, that a man with an artificial upper and an artificial lower, has reasons to congratulate himself as far as his pocket and nerves are concerned: he knows that he has escaped from your bills and your ills.

